# Tricking *Arthrinium malaysianum* into Producing Industrially Important Enzymes Under 2-Deoxy D-Glucose Treatment

**DOI:** 10.3389/fmicb.2016.00596

**Published:** 2016-05-13

**Authors:** Soumya Mukherjee, Mathu Malar Chandrababunaidu, Arijit Panda, Suman Khowala, Sucheta Tripathy

**Affiliations:** ^1^Drug Development Diagnostic and Biotechnology Division, Council of Scientific and Industrial Research-Indian Institute of Chemical BiologyKolkata, India; ^2^Structural Biology and Bioinformatics Division, Council of Scientific and Industrial Research-Indian Institute of Chemical BiologyKolkata, India

**Keywords:** 2-deoxy d-glucose, anti-metabolite, Carbon catabolic repressor, Carbohydrate active enzymes, Cell wall degrading enzymes, unfolded protein response

## Abstract

This study catalogs production of industrially important enzymes and changes in transcript expression caused by 2-deoxy D-glucose (2-DG) treatment in *Arthrinium malaysianum* cultures. Carbon Catabolite Repression (CCR) induced by 2-DG in this species is cAMP independent unlike many other organisms. Higher levels of secreted endoglucanase (EG), β-glucosidase (BGL), β-xylosidase (BXL), and filter paper activity assay (FPase) enzymes under 2-DG treatment can be exploited for commercial purposes. An integrated RNA sequencing and quantitative proteomic analysis was performed to investigate the cellular response to 2-DG in *A. malaysianum*. Analysis of RNASeq data under 2-DG treated and control condition reveals that 56% of the unigenes do not have any known similarity to proteins in non-redundant database. Gene Ontology IDs were assigned to 36% of the transcripts (13260) and about 5207 (14%) were mapped to Kyoto Encyclopedia of Genes and Genomes pathway (KEGG). About 1711 genes encoding 2691 transcripts were differentially expressed in treated vs. control samples. Out of the 2691 differentially expressed transcripts, only 582 have any known function. The most up regulated genes belonged to Pentose Phosphate Pathways and carbohydrate degradation class as expected. In addition, genes involved in protein folding, binding, catalytic activity, DNA repair, and secondary metabolites were up-regulated under 2-DG treatment. Whereas genes encoding glycosylation pathways, growth, nutrient reservoir activity was repressed. Gene ontology analysis of the differentially expressed genes indicates metabolic process (35%) is the pre-dominant class followed by carbohydrate degradation (11%), protein folding, and trafficking (6.2%) and transport (5.3%) classes. Unlike other organisms, conventional unfolded protein response (UPR) was not activated in either control or treated conditions. Major enzymes secreted by *A. malaysianum* are those degrading plant polysaccharides, the most dominant ones being β-glucosidase, as demonstrated by the 2D gel analysis. A set of 7 differentially expressed mRNAs were validated by qPCR. Transmission electron microscopy analyses demonstrated that the 2-DG treated cell walls of hyphae showed significant differences in the cell-wall thickness. Overall 2-DG treatment in *A. malaysianum* induced secretion of large amount of commercially viable enzymes compared to other known species.

## Introduction

Endophytes represent a promising group of organisms, as they are mostly untapped reservoirs of metabolic diversity. They are often able to degrade cellulose and produce an extraordinary diversity of metabolites (Gianoulis et al., 2012). *Arthrinium sp*. are endophytes grown in diverse ecological niches, also known to produce bioactive compounds that have pharmacological and medicinal applications (Crous and Groenewald, 2013). The first *Arthrinium sp., A. saccharicola* was isolated from *Saccharum officinarum* and was later reported from various habitats such as plants, air and seawater (Miao et al., 2006). *A. malaysianum* is a known contaminant on *Termitomyces* cultures (Sawhasan et al., 2012) and in the present study, this organism is isolated from *Termitomyces* mycellial mats. Reports of several other endophytes belonging to *Arthrinium sp*. (*A*. *arundinis, A. saccharicola)* isolated from marine brown algae *Sargassum sp*. or from a mixed culture of *Termitomyces* isolated from termite comb, displayed considerable cellulolytic activities along with antifungal and antioxidant activity (Hong et al., 2015). *Arthrinium saccharicola, Arthrinium affsacchari, Arthrinium phaeospermum* isolated from *Miscanthus sp*. are known to produce industrially important enzymes (Shrestha et al., 2015). However, protein secretory pathways and the spectrum of proteins natively secreted by this fungus to the extracellular medium still remain virtually unexplored. The production and storage of several secondary metabolites from *Arthrinium sp*. have been reported earlier. Bostrycin, ergosterol, succinic acid, various phenolic compounds and antibiotics such as 1-threo-β-hydroxiaspartic acid are some of the known pharmaceutically important products from these genera. Papulacandins, with inhibitory and antifungal activity against bacteria, molds, yeasts, and fungi are also some of the most commonly found secreted products from this group (Traxler et al., 1980; Larrondo et al., 1996; Aissaoui et al., 1998; Adelantado et al., 2005; Calvo et al., 2005).

Carbohydrates are the primary and preferred source of metabolic carbon for most organisms and are used for generating energy and producing biomolecules (de Sousa Lima et al., 2014). The gene Mig1/CreA/CRE1, a zinc-finger transcription factor conserved in most fungal species mediates carbon catabolite repression (CCR) in presence of easily metabolized carbohydrates such as glucose (Ruijter and Visser, 1997). Relief of CCR by glucose depletion or deletion of creA was found to activate expression of genes involved in cell wall-degrading enzymes (CWDEs). Besides being required for CWDE gene expression, alleviation of CCR is also needed for the activation of alternative metabolic pathways like ethanol assimilation and virulence. These proteins that are responsive to carbon catabolite (de) repression, are generally soluble. CAZymes are released under CC (de) Repression. Low molecular weight carbohydrates can act as inducers for the expression of other genes encoding CAZymes, either working individually or together (Delmas et al., 2012). In *Neurospora crassa*, a number of genes that encode cellulolytic and pectinolytic enzymes show increased transcription levels upon carbon starvation as well as during early exposure to cellulose or pectin (Coradetti et al., 2012; Benz et al., 2014).

In recent years, high throughput next generation sequencing technologies like Roche, Illumina, and Applied Biosystems SOLiD, Ion Torrent are increasingly being used for non-model organisms or where reference genome is not available. Several investigations have been successfully carried out using these techniques lately (Zhou et al., 2014). Recently several *de novo* transcriptome sequencing using Illumina platform has been reported in species such as Safflower (Lulin et al., 2012), *Anopheles* (Crawford et al., 2010); *Eucalyptus* (Mizrachi et al., 2010); making it an attractive option for researchers.

In this study, 2-deoxy D-glucose was used in the growth medium as metabolite for down regulating carbon catabolic repression in *A. malaysianum* followed by induction of CWDE. Glucose is a major structural precursor in filamentous fungi as the dominant monosaccharide component for cell wall β-glucans. When glycolysis is inhibited by 2-DG in aerobic condition, cells continue to produce ATP using alternative energy sources, i.e., amino and fatty acids metabolism. Pentose Phosohate Pathway plays an important role in the production of proteins and therefore this pathway is expected to be relevant especially in an organism with an efficient protein production system (Wick et al., 1957; Chen and Gueron, 1992; Limón et al., 2011).

This is a first of its kind study in endophytic fungus *A. malaysianum* cataloging the metabolic and transcriptional changes taking place under 2-DG regulation. This study further substantiates if 2-DG can be used as a precursor for production of commercially applicable enzymes.

## Materials and methods

2, 2-Diphenyl-1-pycrylhydrazyl, glucose 6-P dehydrogenase (EC 1.1.1.49), p-nitrophenyl-β-D-glucopyranoside (pNPG), CM-Chitin-RBV substrates, D-(+)-glucose, laminarin, 2-deoxy-Dglucose, fructose-1,6-bisphosphate, NADP, ATP, NADH, D-L-isocitrate, phenyl hydrazine-HCl, cystein-HCl, EDTA, cAMP Enzyme Immunoassay Kit (Cat no 100 CA201) were purchased from Sigma Chemical Company, USA. Other chemicals of analytical grade were purchased locally.

### Growing cultures in control and treated conditions

*A. malaysianum* was isolated as a contaminant from the mat of *Termitomyces clypeatus* MTCC 5091 and cultured in potato dextrose agar. The mycelia of *A. malaysianum* was later transferred to 100 ml of growth media in 500 ml shake-flasks containing (%, w/v), Glucose-5, Malt extract-5, Potato extract-20%. Apart from this, other components and micronutrients such as ammonium dihydrogen phosphate 2.5, (%, w/v) of CaCl2.2H2O, 0.037; KH2PO4, 0.087; MgSO4.7H2O, 0.05; Boric acid,0.057; FeSO4.7H2O, 0.025; MnCl2.4H2O, 0.0036; NaMoO4.4H2O, 0.0032; ZnSO4.7H2O 0.03 was added to the culture at pH 5.0, and incubated at 30°C under shaking condition (250 rpm) in orbital shaker. The mycelia mats were harvested by filtration through a double-layered muslin cloth and washed several times with sterile distilled water. The mycelia mats were then stored in −80°C (with excess liquid squeezed out), for 18s RNA sequence extraction. For determination of dry weight, samples collected at various time intervals were centrifuged at 9000 × g for 30 min, and the resulting supernatant was filtered through a 0.45 μ (Millipore). The dry weight of mycelia were measured after repeated washing of the mycelial pellets with distilled water and drying overnight at 70°C. The biomass concentration was measured as grams of dry weight.

For studying 2-DG response on the cultures, mycelium of *A. malaysianum* was added to the media containing a single carbon source (1% of glucose or cellobiose). pH of the medium was adjusted to 5.0. The cultures were grown under shaking conditions at 30 ± 2°C for requisite number of days. A separate set containing the above media ingredients along with addition of 2-DG (0.05%) was also done simultaneously.

### Free-radical scavenging activity assay

Each culture was incubated for 7 days and filtered to separate the mycelium from the media. 100 mg wet mycelial mats were extracted and thoroughly washed with distilled water and homogenized on ice, for 5 mins. The mycelial suspension was centrifuged at 15,000 rpm for 20 min at 4°C. The pellet was resuspended in distilled water and sonicated on ice 4 times for 1 min using ultrasonic sonicator at full amplitude. The suspension was centrifuged at 8000 rpm for 10 min at 4°C to precipitate the coarse particle. The precipitate was then washed, air dried and stored at 4°C. The antioxidant activity of aqueous fungal extracts and the standards were assessed on the basis of the radical scavenging effect of the stable DPPH (2, 2-Diphenyl-1-pycrylhydrazyl; Leung et al., 2006). The DPPH, a stable free radical with the characteristic absorption at 517 nm, was used to study the radical scavenging effects on the fungus. The image of hyphae was captured by using light microscopy.

β-Glucosidase (E.C.3.2.1.21) assay was carried out in the reaction mixture containing 2 mM pNPG as substrate in 0.1 M sodium acetate buffer at pH 5.0 and an appropriate amount of enzyme. One unit of β-Glucosidase activity was defined as the amount of enzyme needed to liberate 1 μM of p-nitrophenol (pNP) per minute under the standard assay conditions. β-Xylosidase (E.C.3.2.1.37) was assayed using a similar method with p-nitrophenyl-β-D-xylopyranoside (pNPX) as substrate. Xylanase was assayed using birch wood xylan as substrate. The solution of 0.9 ml xylan (1%, w/v) and the 0.1 ml of enzyme at appropriate dilution were incubated at 50°C for 10 mins. The reducing sugar was determined by the DNS method at 540 nm with xylose as standard. Filter paper degrading activity (FPA) was assayed according to the IUPAC method using 1 × 6 cm (50 mg) Whatman No.1 filter paper strips as a substrate.

### Determination of enzyme activities

The extracellular β-1, 3-glucanase, β-glucosidase, and chitinase activities were measured using laminarin, p-nitrophenyl-β-D-glucose and CM-Chitin-RBV substrates, respectively. cAMP assay was done according to the technical bulletin provided by the cAMP Enzyme Immunoassay Kit. Hexokinase activity assay was based on the reduction of NADP+ through a coupled reaction with glucose-6-phosphate dehydrogenase (10U ml^−1^) in 1 ml reaction mixture containing Tris–HCl buffer (500 mM, pH 7.5), NADP (6.5 mM), ATP (10 mM), MgCl2 (70 mM), and 100 mM glucose. Increase in absorbance at 340 nm was determined spectrophotometrically at 37°C and activity of the enzyme (U ml^−1^) was measured using the value of molar extinction coefficient of the liberated product (€ = 6.22 mM^−1^ cm^−1^). The phosphoglucose isomerase reaction mixture contained 0.4 mM NADP and 0.9 U ml^−1^ glucose-6-phosphate dehydrogenase with 20 mM fructose-6-phosphate as the substrate. Fructose-1,6-bisphosphatase activity was determined in 1 ml assay mixture containing fructose-1,6-bisphosphate (0.8 mM), Tris–HCl buffer (100 mM, pH 7.5), EDTA (2.4 mM), MgCl2 (74 mM), glucose-6-phosphate dehydrogenase (0.7 U ml^−1^) and phosphoglucose isomerase (3.5 U ml^−1^). Isocitrate Lyase activity was measured by adding 200 μl-_*D*_-_*L*_-isocitrate (10 μmol) in 1 ml assay mixture containing KH_2_PO_4_ (200 μmole, pH 6.8), phenyl hydrazine-HCl (10 μmol), cystein- HCl (6 μmol) and MgCl2 (15 μmol). The glucose-6-phosphate dehydrogenase reaction mixture contained 0.4 mM NADP with 5 mM glucose-6-phosphate (Kuby et al., 1974).

### Total RNA isolation from control and experimental fungus samples

Total RNA was isolated from 100 mg of tissue using Xcelgen Plant RNA Miniprep kit. The quality of RNA was analyzed on 1% denaturing agarose gel. The paired-end cDNA sequencing libraries were prepared for both samples, separately using Illumina TruSeq RNA Library Preparation Kit. Library preparation was started with mRNA fragmentation followed by reverse transcription, second-strand synthesis, pair-end adapter ligation, and finally ended by index PCR amplification of adaptor-ligated library. Library quantification and qualification was performed on Caliper LabChip GX using HT DNA High Sensitivity Assay Kit.

### High throughput transcript sequencing

Sequencing was done using Illumina Hiseq platform (Paired end, 101 bases long) generating 50.6 million paired-end reads (12.4 Gbp) from mycelium (control) and 77.14 million reads (18.4 GB) from 2-DG treated samples. Sequences were assembled separately for control and treated samples as well as together generating merged assembly (~128 million reads after merging 50.6 million control reads and 77.14 million 2-DG treated reads). Assembly was done using several assemblers such as Trinity (Grabherr et al., 2011), Ray (Boisvert et al., 2010), Mira (Chevreux et al., 2004), and ABYSS (Birol et al., 2009). Annotation of the assembled transcripts was carried out using Blast (Altschul et al., 1990), KEGG (Kanehisa and Goto, 2000), Interproscan (Jones et al., 2014), Gene Ontology (Conesa et al., 2005) etc. The expression levels of assembled unigenes/transcripts were measured using fragments per kilobases per million reads (FPKM) method. We mapped treated and control assemblies back into merged assembly for annotation consistency using BLAT (Kent, 2002).

### Generating CDS from unigenes using codon preference

The identification of coding sequences (CDS) from expressed transcripts still remains a challenge. Although many methods exist, a modified log likelihood approach based on codon preference (Staden and McLachlan, 1982) works best. Initially, we generated a codon usage table using CUSP from EMBOSS (Rice et al., 2000), where the entire transcript set was used for generating training dataset. Then we iterated this process several times using the modified outputs from the previous dataset and extracted codon preference data from that and used them for training in the subsequent prediction. Fourth iteration produced the best CDS that were subsequently used for analysis.

### qPCR protocols

Based on their functional importance, seven genes were selected for validating the results of the RNA-Seq analysis. The primers for these genes were designed with Primer Express 3.0 software (Applied Biosystems). Total RNA was extracted from both sets of independent cultures grown in absence (control) and presence (treated) of 2-DG in cellobiose media as described above, and then converted to cDNA by random priming. PCR reactions were run in four replicates. The transcription level of genes was determined according to the 2^−Δ*ΔCT*^ method, using actin as a reference gene for the normalization of gene expression levels.

### Second dimension gel electrophoresis (2DGE) and protein visualization, MALDI-TOF MS/MS

Tri Caboxylic Acid precipitated protein (120 μg,) were loaded onto pH 3-10, 7 cm IPG strips with active rehydration for 12 h in rehydration buffer [7 mol/l urea, 2 mol/l theorem, 4% (w/v) CHAPS, 65 mmol/l DTT, 0.2% (v/v) IPG buffer (pH 3–10), trace bromophenol blue]. Isoelectric focusing (IEF) procedure was performed at 25°C using the following setting: S1 linear 250 V 30 min, S2 rapid 500 V 30 min, S3 rapid 1000 V 1 h, S4 linear 10,000 V 4 h, S5 rapid 10,000 V 60 kVh, S6 rapid 500 V 24 h. The second-dimensional SDS-PAGE was performed with 10% polyacrylamide gel in Protein cell IEF (BIO-RAD, USA). The parameter of electrophoresis: 100 V 30 min, 180 V 6 h. The gels were stained in the base of colloidal Coomassie Brilliant Blue G-250. The gel images were imported into the PDQuest 8.0.1 (Bio-Rad, Hercules CA, USA) image software for analysis. The generated peak-lists were searched against fungal database using the MASCOT software package (Version 2.1, Matrix Sciences, U.K.; www.matrixscience.com).

### Study of *A. malaysianum* ultra-structure by transmission electron microscopy (TEM)

Ultra structural damage of 2-DG treated mycelia was ascertained through TEM studies carried out with a TECNAI SPIRIT (FEI, The Netherlands) instrument having an accelerating voltage of 60 kV. Culture aliquots were centrifuged at 10,000 rpm for 10 min; the mycelial pellet was fixed with 2.5% glutaraldehyde in 0.25 M phosphate buffer (pH 7.2) for 24 h, washed thrice with the same buffer and post fixed with 1% w/v osmium tetroxide (2 h). This was followed by 2 h incubation with 0.5% w/v aqueous uranyl acetate solution followed by dehydration using a graded alcohol series (from 50 to 100% v/v) and embedding in spurr. Mycelial thin sections (60 nm) were cut with a diamond knife (DUPONT, Germany) in a UC6 Leica Ultra microtome (Germany) and mounted on copper grids before examination by TEM.

## Results

### Significant increase in the production of cellulolyitc enzymes under 2-deoxy D-glucose treatment

**Figure 1:**
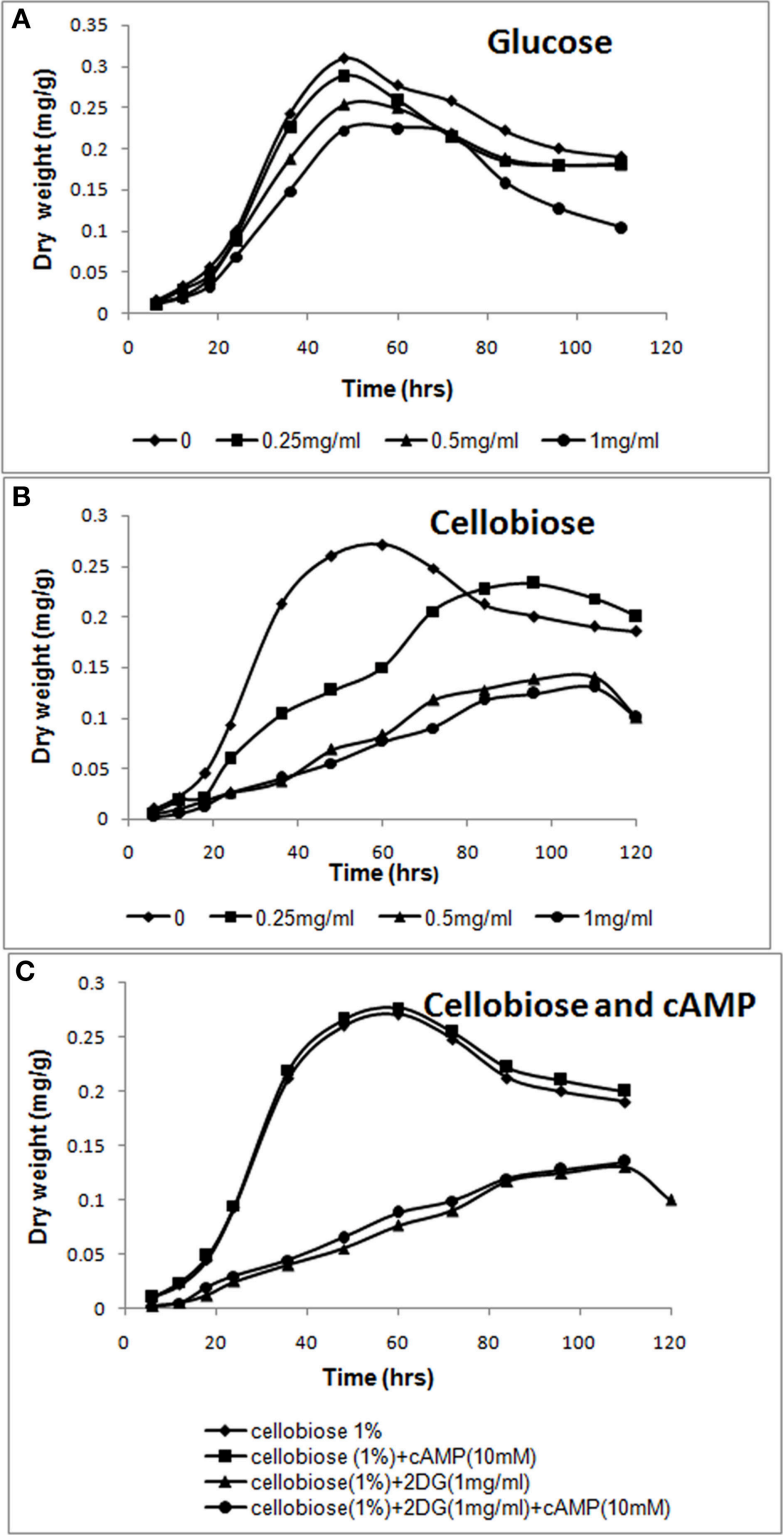
**cAMP-independent CCR by 2-deoxyglucose. (A,B)** Increasing amounts of 2-deoxyglucose (0–1 mg/ml) was added to *A. malaysianum* cultures supplemented with glucose (1%) or cellobiose (1%). **(C)** cAMP and 2-deoxyglucose were added to *A. malaysianum*. cultures supplemented with cellobiose (1%), cellobiose + cAMP (10 Mm), and cellobiose+2DG (1 mg/ml) and + cellobiose+ 2DG (1 mg/ml) +cAMP (10 mM).

*A. malaysianum* was grown in cellobiose and glucose medium (1%) with (treated) and without 2-Deoxy D-Glucose it (control) (Figure 1). To investigate the potential cellulolytic enzyme activities of the fungus secreted in cellobiose medium, the filter paper activity assay (FPase), endoglucanase (EG), β-glucosidase (BGL), Xylanase (X) and β-xylosidase (BXL) activities were measured. This data was compared with another *Arthrinium sp*. e.g., *A. saccharicola* and other cellulolytic fungi such as *Penicillium* sp., *Aspergillus* sp., *Trichoderma* sp. Significantly improved activity of FPase (0.67U/ml), BGL (4.06U/ml), EG (1.38U/ml), Xylanase (3.02U/ml), and BXL(0.53U/ml) was detected in *A*. *malaysianum* compared to 0.39 U/ml FPase, 1.04 U/ml BGL, 0.38 U/ml EG and negligible BXL with no Xylanase activity in *A. saccharicola* (Table 1A). *In vitro* addition of 2-DG in cellobiose medium resulted in further increase of β-glucosidase (14.02U/ml), endoglucanase (4.02 U/ml), β 1, 3 glucanase (1.78 U/ml), chitinase (1.75 U/ml) activity by several folds (Table 1B).

**Table 1A:** **Quantitative comparison of enzyme activities of ***A. saccharicola*** KUC21221 with ***A. malaysianum*** and other terrestrial fungi**.

**Enzyme**	**Strains**	**Substrate**	**Culture conditions**	**Activity (U/mL)**	**References**
FPase	*A. saccharicola* KUC 21221	cellulose	Shake flask, 25°C, 168 h	0.39	Lulin et al., 2012
	*Penicillium echinulatum*	cellulose	Stir fermenter, 37°C,192 h	0.27	Crawford et al., 2010
	*TrichodermaQM6a (wild type)*	lactose	Shake flask, 28°C, 120 h	0.19	Ref
	*A. malaysianum*	cellobiose	Shake flask, 28°C, 96 h	0.67	This study
EG	*A. saccharicola* KUC 21221	cellulose	Shake flask, 25°C, 168 h	0.39	Lulin et al., 2012
	*Hypoxylon oceanicum*	cellulose + Filter paper	Shake flask, 25°C, 360 h	0.04	Mizrachi et al., 2010
	A. *malaysianum*	cellobiose	Stir fermenter, 28 °C, 96 h	1.38	This study
BGL	*A. saccharicola* KUC 21221	cellulose	Shake flask, 25 °C, 168 h	1.04	Lulin et al., 2012
	*Aspergillus niger*	cellulose	Shake flask, 28 °C, 168 h	1.02	Basselin-Eiweida and Kaneshiro, 2001
	*A. malaysianum*	cellobiose	Shake flask, 28 °C, 96 h	4.06	This study
BXL	A. *saccharicola* KUC 21221	cellulose	Shake flask, 25 °C, 168 h	nil	Lulin et al., 2012
	*A. malaysianum*	cellobiose	Shake flask, 28 °C, 96 h	0.53	This study

*FPase, filter paper activity; EG, endoglucanase; BGL, β-glucosidase; BXL, β-xylosidase*.

**Table 1B:** **Characteristic properties of ***A. malaysianum*** cultures: Mycelia from exponential phase of ***A. malaysianum*** (approximately after 24 h) were washed and transferred into either glucose containing 2-deoxy D-glucose or cellobiose containing 2-deoxyglucose or in minimal medium containing just cellobiose**.

**Property**	**Glucose +2-DG**	**Cellobiose +2-DG**	**Cellobiose control**
Carbon content(g/l)	10±0.001	10±0.001	10±0.001
β-glucosidase (U/ml)	2±0.3	14±1.23	4±0.5
β,1,3 glucanase (U/ml)	0.2±0.02	1.78±0.05	0.5±0.23
Radical scavenging activity	2.5±0.001	8.1±0.001	3.5±0.11
Superoxide content	0.025±0.01	0.066±0.01	0.047±0.35
Change in Dry Cell Mass (g/l)	0.5±0.33	1.5±0.5	3.0±1.2
Ph	5±0.001	4.5±0.002	5±0.01
Chitinase (U/ml)	0.4±0.2	1.75±0.7	0.39±0.43
Endoglucanase (U/ml)	0.22±0.01	4±0.22	1.3±0.1
Hyphae fragmentation	no	yes	no
Pigment formation	no	yes	no

*Mean ± SD values calculated from five independent experiments are presented*.

### Increased secretion of β-glucosidase in 2-DG treated condition

Cellobiose consumption and β-glucosidase activity were monitored during cultivation of the *A. malaysianum* strains in cellobiose (1%) + 2-DG (0.5 mg/ml) media.

**Figure 2:**
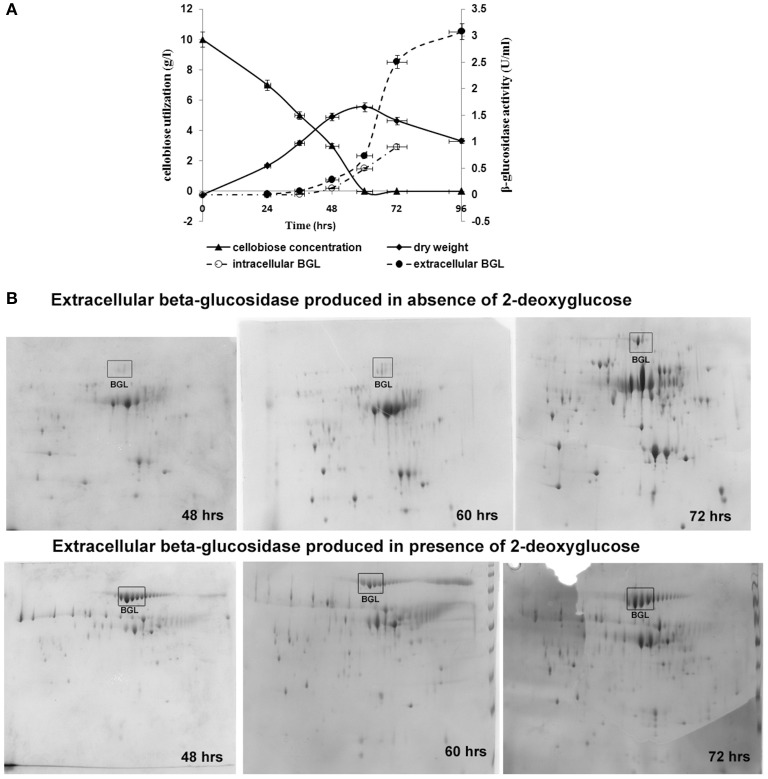
**Growth, carbon consumption, and synthesis of β-glucosidase in ***A. malaysianum*** in presence of 0.5 mg/ml of 2DG. (A)** Time dependent profile of fungal growth, cellobiose utilization, and kinetics of intracellular and extracellular β-glucosidase production, **(B)** 2D Gel electrophoresis analysis of the secreted proteins of *A. malaysianum* at three different hours. Extracellular β-glucosidase secreted into the culture medium in absence **(A,B)** in presence of 2DG (0.5 mg/ml) at 48, 60, and 72 h is highlighted as (Muller et al., 2015).

**Figure 3:**
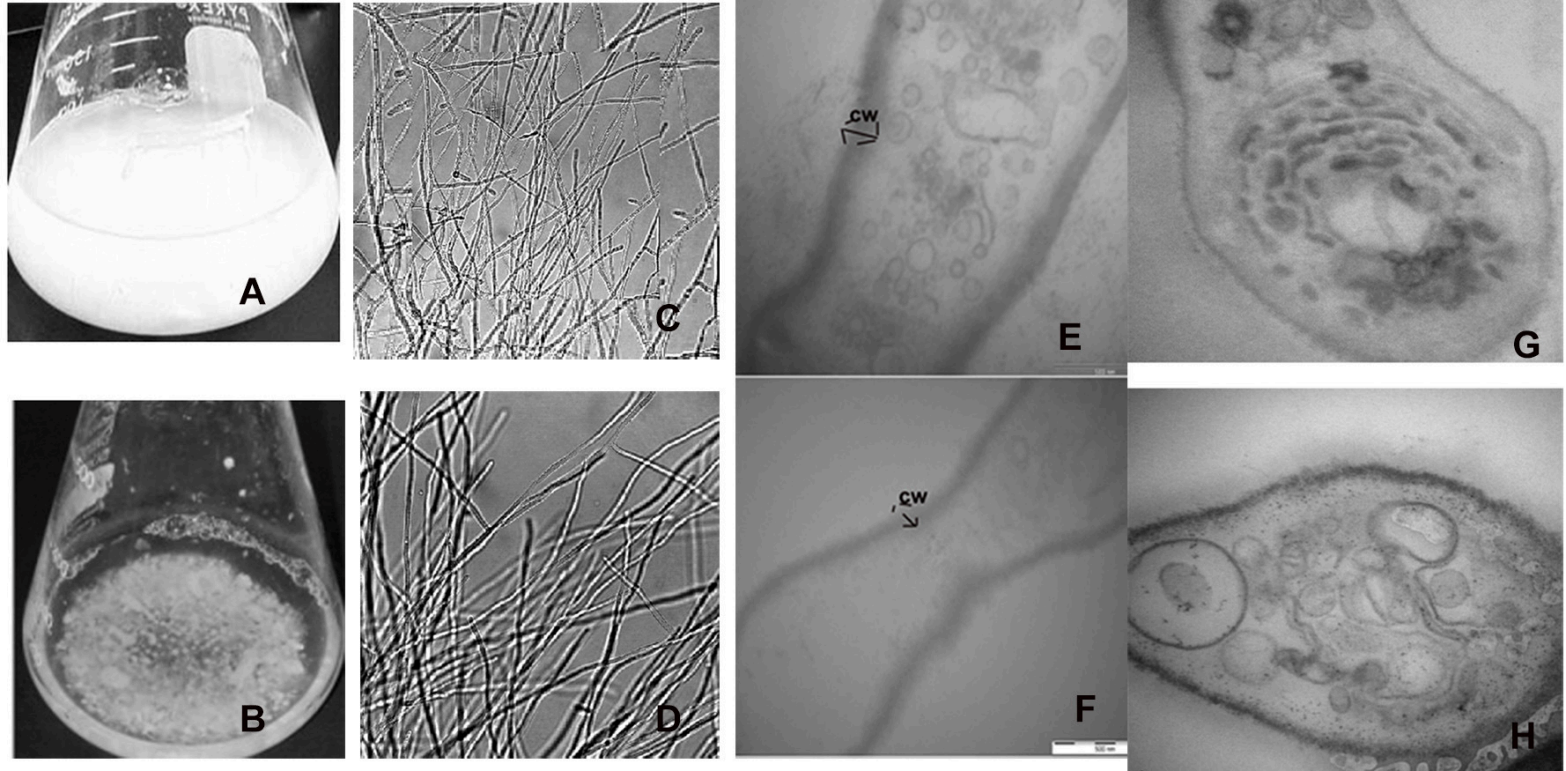
**Formation of aberrant hyphae structure in treated mycelia cultured. (A)** Cellobiose cultures in absence of 2DG and **(B)** in presence of 2DG after 48 h. **(B)** Morphological changes in the hyphae of *A. malaysianum* grown in **(C)** absence and **(D)** presence of 2-deoxyglucose as observed in simple microscope, Longitudinal **(E,F)** and transverse section **(G,H)** of the hyphae in absence **(E,G)** and presence **(F,H)** of 2DG.

In presence of 2-DG, intracellular β-glucosidase activity appeared within 48 h of cultivation (0.067 U/ml), when 40% of the cellobiose still remained in the culture medium. Maximum activity of 1.0 U/ml was achieved at 72 h after which no intracellular β-glucosidase was detected. Extracellular β-glucosidase activity appeared after 48 h and increased gradually until 96 h and attained a maximum value 3 U/ml. Rise in extracellular β-glucosidase was observed till 96 h (Figure 2A). In the attempt to identify enzymatic reactions associated with the cell walls which correlate with high release of extracellular β-glucosidase into the medium, the cell wall bound activities of the hydrolytic enzymes like β,1-3 glucanase, chitinase, endoglucanase were measured during growth on glucose +2-DG, cellobiose +2-DG and cellobiose media (Table 1B; Figure 3). The β, 1-3 glucanase activity was highest in cellobiose+2-DG media (1.78 U/ml) and lowest in glucose +2-DG media (0.2 U/ml). Similarly chitinase and endoglucanase activity was highest in cellobiose+2-DG medium (1.7 and 4U/ml, respectively).

Water extracts of mycelia of both treated (cellobiose +2-DG) and control (cellobiose) of *A. malaysianum* were subjected to possible antioxidant activity by the DPPH free radical scavenging method. The model of scavenging the stable DPPH radical is widely used to evaluate antioxidant activities over a relatively short time compared to other methods. Scavenging effect of mycelia extracts on DPPH radicals was found to be concentration dependent. Free radical scavenging effect of 8.1% at a lower concentration of 2 mg/ml of mycelia extracts was achieved in 2-DG treated mycelium as shown in Table 1B.

### Proteome analysis of β-glucosidase secretion

Total proteins secreted to extracellular milieu during cultivation of *A. malaysianum* in treated (cellobiose in presence of 2-DG) and control (cellobiose in absence of 2-DG) medium was extracted at different time interval (48, 60, 72 h) and was analyzed by 2-DGE analysis, (Figure 2B). Most of the protein spots were concentrated in pI range 4–7 and between molecular mass of 30 and 120 kDa. It was clear from the gel images that extracellular β-glucosidase in absence of 2-DG was produced after 60 h of cultivation whereas extracellular β-glucosidase in presence of 2-DG was produced within 48 h and secreted into the medium (Figure 2B).

### Transcriptome assembly and annotation

**Figure 4:**
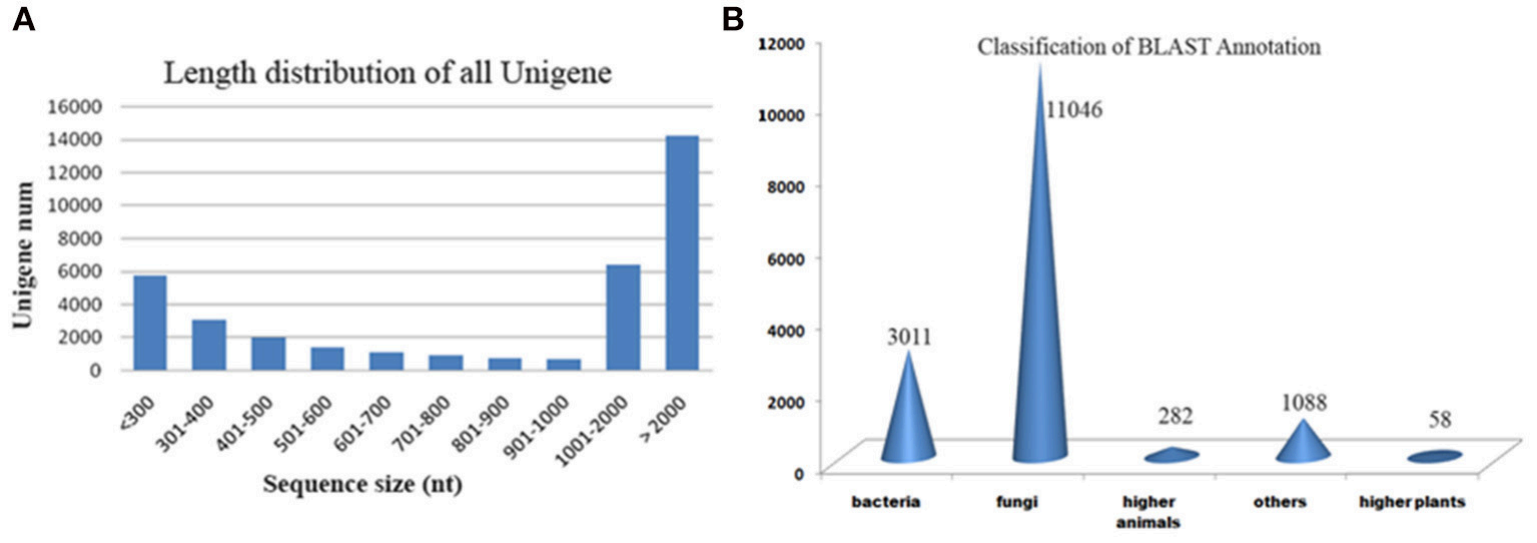
**(A)** Length distribution of transcripts. **(B)** Blast results of *A. malaysianum* against non-redundant database.

We generated deep transcriptome sequences with an approximate coverage of 250X from control and 2-DG treated samples using Illumina platform. Transcript reads from control and treated samples were assembled separately and together (merged assembly) for analyzing differential expression. Out of the several assemblers used, Trinity assembler (Grabherr et al., 2011) generated the best results. Further, downstream data analysis was carried out using merged assembly. We used dinucleotide relative abundance (Gentles and Karlin, 2001) to calculate possible contaminants and removed about 84 sequences from the merged assembly. Final assembly resulted in 21192 genes (termed as unigenes) coding for 27252 transcripts from control samples, 22372 unigenes coding for 32982 transcripts from 2-DG treated samples and 22847 unigenes encoding 36248 transcripts from merged samples (Table 2). The length distribution of the assembled transcripts are represented in Figure 4A.

**Table 2:** **Reads pre-processing and transcript assembly statistics using Trinity**.

**Library**	**Total Reads^#^**	**High quality reads^#^**	**Equalized high quality reads^#^**	**Number of assembled Transcripts**	**Corresponding Number of genes**	**N50, Average transcript length, GC content of final assembly**
Control	25329795 (R) +	22273695 (R) +	20744877 (R) +	27252	21192	2414, 1296.79,
	25329795 (F)	22737139 (F)	20744877 (F)			54.13%
		**80%**	**70%**			
Treated	38573096 (R) +	36395969 (R) +	34885064 (R) +	32982	22372	2770, 1580.90,
	38573096 (F)	36329694 (F)	34885064 (F)			53.8%
		**80%**	**70%**			
Merged	63902891 (R) +	55629941 (R) +	55629941 (R) +	36248	22847	3659, 2055.82,
	63902891 (F)	55629941 (F)	55629941 (F)			53.5%
		**80%**	**80%**			

# *Total reads indicate the number of raw reads. High quality reads are the quality filtered reads obtained from the raw reads. Equalized reads are the pairs of high quality reads available. Since in many cases, only one of the pair is good quality where as the other pair is poor quality. In that case, the pair of reads are removed*.

Putative functions were assigned to 44% (16,096) transcripts based on their similarities to non redundant database (Supplementary Table 1). Out of which, 152 (0.4%) transcripts matched with ribosomal genes. A significantly large number of orthologs were detected with Ascomycetes fungus *Eutypa* followed by Bacteria (Figure 4B).

Taxonomy analysis *A. malaysianum* unigenes indicated that it had 55% sequence similarity with *Eutypa lata* UCREL1 (3588) species, followed by *Colletotrichum higginsianum* (1499), *Gaeumannomyces sp*. (304), *Glomerella* s*p*. (546), *Thielavia sp* (312), *Togninia sp*. (984), respectively. The secretome analysis was also done to identify the diverse array of secretory proteins of *A. malysianum* (Supplementary Table 2). A large part of the secretome was classified as hypothetical proteins followed by phosphorylation related proteins, Glycosyl hydrolases and transferase. The major gene ontology processes in *A. malaysianum* were consistent with those of *Eutypa lata, Gaeumannomyces graminis, Thielavia sp*., *Togninia*, and *Colletotrichum* (O'Connell et al., 2012; Jaramillo et al., 2015).

Among the GO classifications, 7351 transcripts were assigned to molecular process; 230 under cellular component and 838 under biological process. Under each of the three main categories (biological process, cellular component and molecular function), “metabolic process,”, “cell,” “cellular process,” “binding,” and “catalytic activity” terms occupied the largest proportion in both the control and experimental samples. We also noticed a high percentage of experimental transcripts from categories of “transporter activity,” “organelle” and “protein binding.” “Nutrient reserve activity,” “viral reproduction” and “protease activity” was more prominent in 2-DG treated transcripts.

## About 7.3% genes are differentially expressed under 2-DG treated conditions

**Figure 5:**
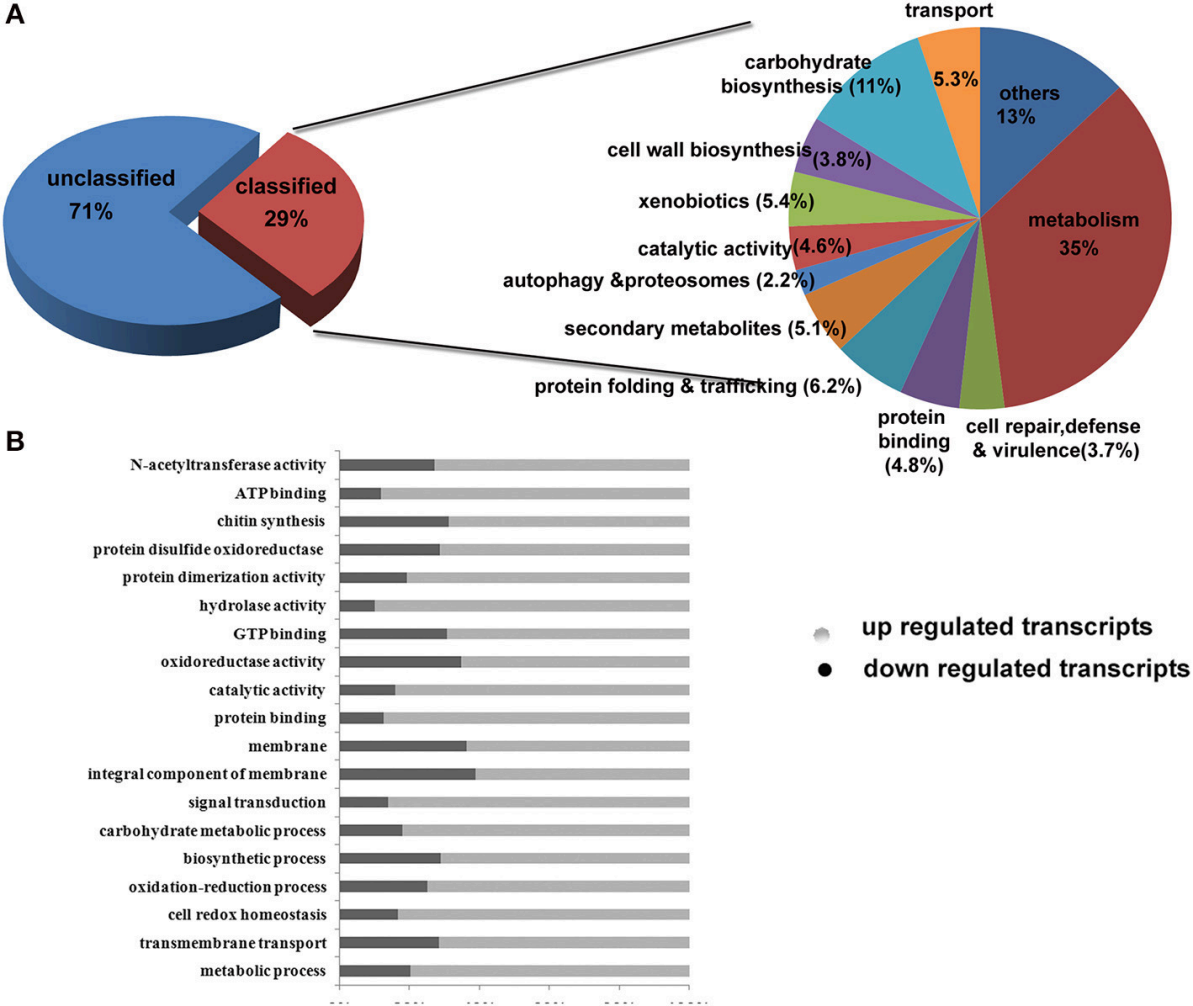
**Classification on the basis of Gene Ontology (GO) functional annotation**.

We used a fold change value of >=2 for estimating differentially expressed genes. A total of 2691 transcripts encoded by 1711 genes were differentially expressed under control and treated conditions. Out of this, 1150 transcripts were down regulated and 1540 transcripts were up-regulated. The list of genes and their individual functions with their KO id's are provided in Supplementary Table 3. Out of the differentially expressed genes, the number of carbohydrate metabolism (11%) genes was the highest in number followed by secondary metabolites (5.1%), transporters (5.3%), binding proteins (4.8%), catalytic activity (4.6%), and cell rescue and virulence genes (3.7%; Figure 5). Differentially expressed transcripts were analyzed further on several key functions such as (i) carbohydrate-active enzymes(CAZymes) and cell wall biogenesis (ii) Secondary metabolism, unfolded protein response and virulence genes iii) metabolic pathways and enzymes, and (iv) Other functions mainly on cellular transporters and cell repair.

### Enhanced expression of cazymes and cell wall homeostasis during 2-DG treatment

Enzymes that break down plant cell wall can be categorized into the following classes: glycoside hydrolases (GH), glycosyl transferases (GT) polysaccharide lyases (PL), carbohydrate esterases (CE), and auxiliary activities (AA). All CAZymes and auxiliary activity enzymes can be attached to carbohydrate-binding modules (CBMs). Our transcriptomic analysis revealed a vast majority of carbohydrate-active enzymes encoded by *A. malaysianum*. About 3447 CAZymes were detected from the merged transcriptome (Supplementary Table 4).

We predicted carbohydrate degrading enzymes and their annotations using CAZymes Analysis Toolkit (CAT) that uses CAZY database (Cantarel et al., 2009) (Table 3; Supplementary Table 4). A core set of 302 genes encoding CAZymes was up-regulated in response to 2-DG. The results show that 16 β-glucosidase (bglX) belonging to GH 1 and 6 glucan 1,3-betaglucosidase (elgA) belonging to GH 5 family was significantly upregulated. Both theses enzymes are involved in cell wall biogenesis/degradation. Other glycosidases upregulated were α-glucosidases (2), β-galactosidases (2), 1 polygalacturonases and 5 chitinases. Beside this, a large number of cytochrome superfamily (CYP5A CYP1A1) and esterase were also upregulated (Table 4). In addition, 30 polyketide related genes, 6 non-Ribosomal peptide along with many bioactive compound producing genes are up-regulated making this fungus an industrially important strain to both biofuel and pharmaceutical industry (Supplementary Table 1).

**Table 3:** **Up- and down-Fold increased in presence of 2DG of differentially expressed genes encoding CAZymes of ***A. malaysianum*****.

**Description**	**ID**	**Family**	**Fold**
α-amylase	ko:K01176	13	−1.36
beta-amylase	ko:K01178	14	1.13
chitinase	ko:K01183	19	3.88
beta-mannosidase	ko:K01192	47	2.1
hexosaminidase	ko:K12373	20	1.4
Mannosyl oligosaccharide α-1,2-mannosidase	ko:K01230	92	−0.238
beta-glucosidase	ko:K05349 ko:K01188	3	9.336
glucan 1,3-beta-glucosidase	ko:K01210	17	2.129
alpha-L-fucosidase 2	ko:K15923	29	3.09
beta-galactosidase	ko:K01190	42	2.41
alpha 1,3-glucosidase	ko:K05546	31	2
pectinesterase	ko:K01051	8	3.7
endoglucanase	ko:K01179	9	5.2
polygalacturonase	ko:K01184	28	6.3
beta-fructofuranosidase	ko:K01193	32	1.01
alpha, alpha-trehalase	ko:K01194	37	2.2
xylan 1,4-beta-xylosidase	ko:K01198	3	4.65
beta-N-acetyl hexosaminidase	ko:K01207	3	2.11
1,4-alpha-glucan branching enzyme	ko:K00700	13	3.42

**Table 4:** **List of some of the important differentially expressed genes**.

**ID**	**Description**	**Log2 (_treated FPKM/control_FPKM)**
***CARBOHYDRATE METABOLISM***		
comp2979_c0_seq1	hexokinase	−3.264
comp7311_c0_seq2	glucan 1,3-beta-glucosidase	4.131
comp9565_c0_seq1	polygalacturonase	10.924
comp9273_c0_seq2	endo-1,4-beta-xylanase	10.399
comp9579_c0_seq12	beta-glucosidase	10.533
comp9570_c0_seq21	ribose 5-phosphate isomerase	9.903
comp5010_c0_seq1	transaldolase	15.125
comp9146_c0_seq11	keto hexokinase	8.888
comp4157_c0_seq1	glycerol 3-phosphatase 1	7.24
comp9393_c0_seq6	fructose-1,6-bisphosphatase I	10.35
comp3867_c0_seq1	glucose 1-dehydrogenase	3.219
comp9566_c0_seq13	succinyl-CoA synthetase alpha subunit	10.218
**OXIDATIVE STRESS**		
comp9406_c0_seq1	short-chain dehydrogenase	1.96
comp8352_c0_seq1	cytochrome P450	2.58
comp1121_c0_seq1	benzoate 4-monooxygenase cytochrome P450	1.20
comp7525_c0_seq2	catalase	-3.009
comp14211_c0_seq1	glutathione S-transferase	3.157
comp9259_c0_seq4	CDK inhibitor PHO81	8.420
**SECONDARY METABOLITES**		
comp16_c1_seq1	lovastatin nonaketide synthase	0.79
comp562251_c0_seq1	polyketide synthase-like protein	2.08
comp7753_c0_seq2	Cytochrome P450	5.155
comp8296_c0_seq1	farnesyl diphosphate synthase	0.91
comp43478_c0_seq1	polyketide synthase	2.70
comp121975_c0_seq1	Putative Monooxygenase FAD-binding protein	0.870
**DEFENSE AND VIRULENCE GENE**		
comp550945_c0_seq1	putative aflatoxin biosynthesis ketoreductase nor-1 protein	0.321
comp10847_c0_seq1	putative aflatoxin b1 aldehyde reductase member 2 protein	0.755
comp529707_c0_seq1	gramicidin synthetase	0.65
comp501600_c0_seq1	toxin HicA	0.53
comp195398_c0	killer toxin subunits alpha beta protein	0.43
comp305955_c0_seq1	putative capsule polysaccharide biosynthesis protein	1.722
comp363556_c0_seq1	Beta-lactamase-like protein 2	2.655
comp25830_c0_seq1	putative parasitic phase-specific protein psp-1 protein	0.11
comp7944_c0_seq7	paxillin	3.259
comp9761_c1_seq28	mitogen-activated protein kinase-activated protein kinase 2	9.226
**PROTEIN FOLDING**		
comp2198_c0_seq1	putative disulfide isomerase protein	1.25
comp2220_c0_seq1	GroES-like protein	0.76
comp9842_c0_seq1	Vacuolar transport chaperonin	2.22
comp4600_c0_seq2	Chaperone and intramolecular catalysts	13.364
comp7807_c0_seq2	proline iminopeptidase	5.202
comp41741_c0_seq1	Tri Peptidyl–peptidase	4.034
comp8729_c0_seq5	DnaJ homolog subfamily A member 2	−4.374
comp7183_c0_seq2	DNA mismatch repair protein MSH6	9.394
comp8338_c0_seq3	DNA replication licensing factor MCM4	10.876
**ANTIOXIDANTS AND XENOBIOTICS**		
comp253010_c0_seq1	antioxidant, AhpC/TSA family,	0.88
comp29016_c0_seq1	l-asparaginase	0.93
comp252656_c0_seq1	1,3-beta-glucanosyltransferase Bgt1	0.54
comp3196_c0_seq1	putative phytoene dehydrogenase protein	2.974
comp5186_c0_seq1	esterase/lipase	3.853
**TRANSCRIPTORS FACTORS**		
comp9593_c0_seq10	bZIP transcription factor	−0.3
comp6086_c0_seq1	C_2_H_2_andC_2_HCzincfinger	1.39
comp12307_c0_seq1	Catabolite repression protein xlnr	0.37
comp9263_c0_seq2	Fungal specific transcription factor	−8.002
comp9471_c0_seq22	GAL4	9.711
**OTHERS**		
comp8381_c0_seq1	3-oxoacyl-[acyl-carrier-protein] reductase FabG	9.721
comp9759_c0_seq61	thromboxane-A synthase	6.743
comp9547_c0_seq26	tyrosyl-tRNA synthetase	5.721
comp6181_c0_seq2	1-acylglycerone phosphate reductase	12.796
comp4188_c0_seq1	fatty acid synthase	9.306
comp9705_c1_seq30	two-component system, NarL family, capsular synthesis sensor histidine kinase RcsC	10.292
comp6442_c0_seq2	Polycyclic aromatic hydrocarbon degradation	9.512
comp12016_c0_seq1	phosphatidylinositol 3-kinase	3.301
**TRANSPORTERS**		
comp8307_c0_seq2	siderochrome-iron transporter	−9.394
comp3213_c0_seq2	putative pantothenate transporter protein	0.49
comp7724_c0_seq1	mfs general substrate transporter	3.8
comp7708_c0_seq2	putative abc transporter family protein	9.691
comp4371_c0_seq2	high-affinity nicotinic acid transporter	2.71
comp3454_c0_seq1	putative high-affinity glucose transporter rgt2 protein	−2.764
comp2836_c0_seq1	putative sugar transporter stl1 protein	1.76
comp515146_c0_seq1	metabolite transporter	2
comp9622_c0_seq24	arabinose-proton symporter	9.097
comp4447_c0_seq1	solute carrier family 27 (fatty acid transporter), member 1/4	9.534
**CELL WALL BIOSYNTHESIS**		
comp8016_c0_seq1	chitinase chi18–5	1.89
comp2874_c0_seq1	snf2 family helicase protein	0.73
comp775_c0_seq1	endochitinase	1.14
comp522682_c0_seq1	chitinase	1.32
comp9583_c0_seq6	putative chitinase 3 protein	2
comp8733_c0_seq1	chitin synthase activator protein	−0.33
comp8721_c0_seq3	putative chitin binding protein	1.99
comp12947_c0_seq1	class 2 chitin synthase	−0.12
comp5851_c0_seq1	putative chitin synthase 8 protein	−1.7
comp779_c0_seq1	wsc domain containing protein	1.56
comp7545_c0_seq1	Hydrophobin	2.1
comp9753_c0_seq19	Cell wall protein PhiA	0.179
comp9145_c0_seq2	Aquaporins	−3.134
comp8599_c0_seq5	yapsin 1	−8.452
**REDOX**		
comp9567_c0_seq7	NADPH2:quinone reductase	8.295
comp10287_c0_seq1	cytochrome c peroxidase	2.804
**GLYCAN BIOSYNTHESIS AND GLYCOSYLATION**		
comp3969_c0_seq1	mannosyl-oligosaccharide alpha-1,2-mannosidase	−4.385
comp7551_c0_seq1	Glycosylphosphatidylinositol(GPI)-anchor biosynthesis	−3.627
comp4180_c0_seq2	alpha-1,3/alpha-1,6-mannosyltransferase	−3.652
comp6776_c0_seq4	mannosyl-oligosaccharide alpha-1,2-mannosidase	9.051
ID	Description	Log2 (_treated FPKM/(control_FPKM)
***CARBOHYDRATE METABOLISM***		
comp2979_c0_seq1	hexokinase	−3.264
comp7311_c0_seq2	glucan 1,3-beta-glucosidase	4.131
comp9565_c0_seq1	polygalacturonase	10.924
comp9273_c0_seq2	endo-1,4-beta-xylanase	10.399
comp9579_c0_seq12	beta-glucosidase	10.533
comp9570_c0_seq21	ribose 5-phosphate isomerase	9.903
comp5010_c0_seq1	transaldolase	15.125
comp9146_c0_seq11	keto hexokinase	8.888
comp4157_c0_seq1	glycerol 3-phosphatase 1	7.24
comp9393_c0_seq6	fructose-1,6-bisphosphatase I	10.35
comp3867_c0_seq1	glucose 1-dehydrogenase	3.219
comp9566_c0_seq13	succinyl-CoA synthetase alpha subunit	10.218
**OXIDATIVE STRESS**		
comp9406_c0_seq1	short-chain dehydrogenase	1.96
comp8352_c0_seq1	cytochrome P450	2.58
comp1121_c0_seq1	benzoate 4-monooxygenase cytochrome P450	1.20
comp7525_c0_seq2	catalase	−3.009
comp14211_c0_seq1	glutathione S-transferase	3.157
comp9259_c0_seq4	CDK inhibitor PHO81	8.420
**SECONDARY METABOLITES**		
comp16_c1_seq1	lovastatin nonaketide synthase	0.79
comp562251_c0_seq1	polyketide synthase-like protein	2.08
comp7753_c0_seq2	Cytochrome P450	5.155
comp8296_c0_seq1	farnesyl diphosphate synthase	0.91
comp43478_c0_seq1	polyketide synthase	2.70
comp121975_c0_seq1	Putative Monooxygenase FAD-binding protein	0.870
**DEFENSE AND VIRULENCE GENE**		
comp550945_c0_seq1	putative aflatoxin biosynthesis ketoreductase nor-1 protein	0.321
comp10847_c0_seq1	putative aflatoxin b1 aldehyde reductase member 2 protein	0.755
comp529707_c0_seq1	gramicidin synthetase	0.65
comp501600_c0_seq1	toxin HicA	0.53
comp195398_c0	killer toxin subunits alpha beta protein	0.43
comp305955_c0_seq1	putative capsule polysaccharide biosynthesis protein	1.722
comp363556_c0_seq1	Beta-lactamase-like protein 2	2.655
comp25830_c0_seq1	putative parasitic phase-specific protein psp-1 protein	0.11
comp7944_c0_seq7	paxillin	3.259
comp9761_c1_seq28	mitogen-activated protein kinase-activated protein kinase 2	9.226
**PROTEIN FOLDING**		
comp2198_c0_seq1	putative disulfide isomerase protein	1.25
comp2220_c0_seq1	GroES-like protein	0.76
comp9842_c0_seq1	Vacuolar transport chaperonin	2.22
comp4600_c0_seq2	Chaperone and intramolecular catalysts	13.364
comp7807_c0_seq2	proline iminopeptidase	5.202
comp41741_c0_seq1	Tri Peptidyl–peptidase	4.034
comp8729_c0_seq5	DnaJ homolog subfamily A member 2	−4.374
comp7183_c0_seq2	DNA mismatch repair protein MSH6	9.394
comp8338_c0_seq3	DNA replication licensing factor MCM4	10.876
**ANTIOXIDANTS AND XENOBIOTICS**		
comp253010_c0_seq1	antioxidant, AhpC/TSA family,	0.88
comp29016_c0_seq1	l-asparaginase	0.93
comp252656_c0_seq1	1,3-beta-glucanosyltransferase Bgt1	0.54
comp3196_c0_seq1	putative phytoene dehydrogenase protein	2.974
comp5186_c0_seq1	esterase / lipase	3.853
**TRANSCRIPTORS FACTORS**		
comp9593_c0_seq10	bZIP transcription factor	−0.3
comp6086_c0_seq1	C_2_H_2_andC_2_HCzincfinger	1.39
comp12307_c0_seq1	Catabolite repression protein xlnr	0.37
comp9263_c0_seq2	Fungal specific transcription factor	−8.002
comp9471_c0_seq22	GAL4	9.711
**OTHERS**		
comp8381_c0_seq1	3-oxoacyl-[acyl-carrier-protein] reductase FabG	9.721
comp9759_c0_seq61	thromboxane-A synthase	6.743
comp9547_c0_seq26	tyrosyl-tRNA synthetase	5.721
comp6181_c0_seq2	1-acylglycerone phosphate reductase	12.796
comp4188_c0_seq1	fatty acid synthase	9.306
comp9705_c1_seq30	two-component system, NarL family, capsular synthesis sensor histidine kinase RcsC	10.292
comp6442_c0_seq2	Polycyclic aromatic hydrocarbon degradation	9.512
comp12016_c0_seq1	phosphatidylinositol 3-kinase	3.301
**TRANSPORTERS**		
comp8307_c0_seq2	siderochrome-iron transporter	−9.394
comp3213_c0_seq2	putative pantothenate transporter protein	0.49
comp7724_c0_seq1	mfs general substrate transporter	3.8
comp7708_c0_seq2	putative abc transporter family protein	9.691
comp4371_c0_seq2	high-affinity nicotinic acid transporter	2.71
comp3454_c0_seq1	putative high-affinity glucose transporter rgt2 protein	−2.764
comp2836_c0_seq1	putative sugar transporter stl1 protein	1.76
comp515146_c0_seq1	metabolite transporter	2
comp9622_c0_seq24	arabinose-proton symporter	9.097
comp4447_c0_seq1	solute carrier family 27 (fatty acid transporter), member 1/4	9.534
**CELL WALL BIOSYNTHESIS**		
comp8016_c0_seq1	chitinase chi18-5	1.89
comp2874_c0_seq1	snf2 family helicase protein	0.73
comp775_c0_seq1	endochitinase	1.14
comp522682_c0_seq1	chitinase	1.32
comp9583_c0_seq6	putative chitinase 3 protein	2
comp8733_c0_seq1	chitin synthase activator protein	−0.33
comp8721_c0_seq3	putative chitin binding protein	1.99
comp12947_c0_seq1	class 2 chitin synthase	−0.12
comp5851_c0_seq1	putative chitin synthase 8 protein	−1.7
comp779_c0_seq1	wsc domain containing protein	1.56
comp7545_c0_seq1	Hydrophobin	2.1
comp9753_c0_seq19	Cell wall protein PhiA	0.179
comp9145_c0_seq2	Aquaporins	−3.134
comp8599_c0_seq5	yapsin 1	−8.452
**REDOX**		
comp9567_c0_seq7	NADPH2:quinone reductase	8.295
comp10287_c0_seq1	cytochrome c peroxidase	2.804
**GLYCAN BIOSYNTHESIS AND GLYCOSYLATION**		
comp3969_c0_seq1	mannosyl-oligosaccharide alpha-1,2-mannosidase	−4.385
comp7551_c0_seq1	Glycosylphosphatidylinositol(GPI)-anchor biosynthesis	−3.627
comp4180_c0_seq2	alpha-1,3/alpha-1,6-mannosyltransferase	−3.652
comp6776_c0_seq4	mannosyl-oligosaccharide alpha-1,2-mannosidase	9.051

Majority of CAZymes belonged to GH (117) and GT (62). Few others were categorized under PL (2), CE (27), CBM(35), and AA (52). A increased set of GH, GT and CBM (22) domains can be regarded as a hallmark of convergent evolution of *A. malaysianum* caused due to mixed culture with *Termitomyces clypeatus*.

### 2-DG triggers expression of virulence associated genes, unfolded protein response and biosynthesis of secondary metabolites

Virulence factors are difficult to define by classical terminology. Intrinsically associated with pathogenicity, such as endotoxin and molecules referred to as modulins (e.g., cell wall polysaccharides and lipids), can contribute to the pathogenic process. Transcriptome analysis *A. malaysianum* in 2-DG treated condition revealed the presence of potential virulence associated genes (Table 4). Some requisite virulence factors, such as the toxins or polysaccharide capsules proteins, beta-lactamase like protein were expressed suggesting the pathogenic nature of the organism in stressed environmental condition. Constitutively expressed components of microorganisms, such as endotoxin and molecules referred to as modulins (e.g., cell wall polysaccharides and lipids), that contribute to the pathogenic process were also expressed.

**Figure 6:**
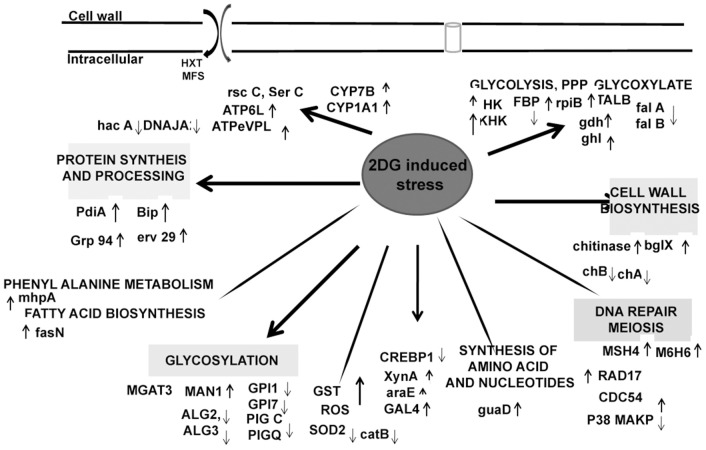
**Proposed changes in the metabolism of ***A. malaysianum*** in 2DG treated conditions**.

**Figure 7:**
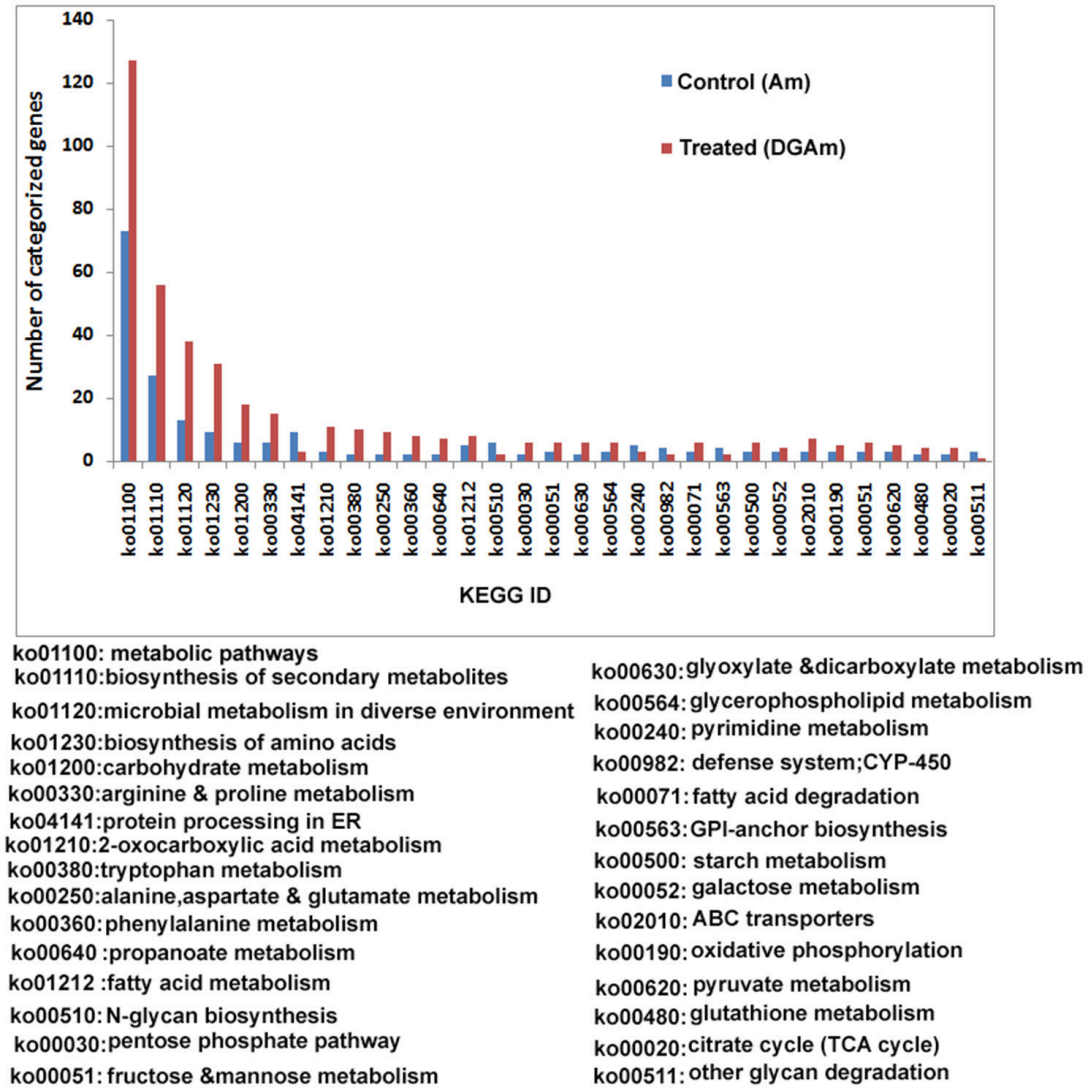
**KEGG analysis of the assembled transcripts**.

**Figure 8:**
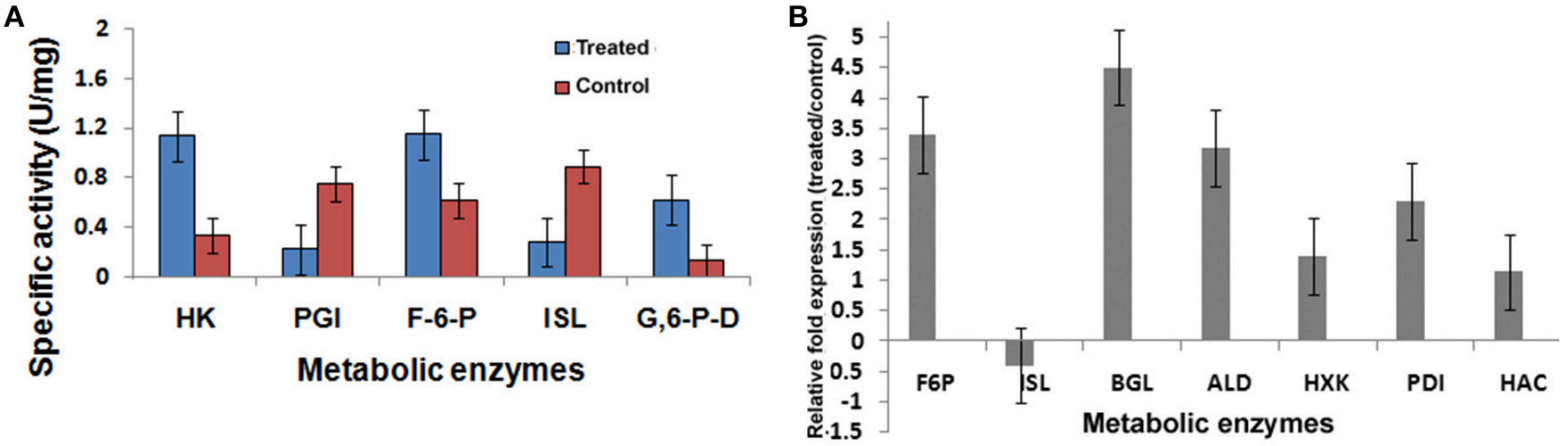
**Metabolic enzyme profile induced by 2DG**. **(A)** Specific activity of Hexokinase (HK), Phosphoglucoisomerase (PGI), Fructose bis phosphate (F6P), Isocitrate lyase (ISL), and Glucose 6 phosphate dehydrogenase (G6PD) and **(B)** Relative fold change of seven genes using qPCR methods. Data represents four replicates with standard error bar.

Surprisingly, several genes with important functions in protein synthesis and trafficking were also upregulated (Table 3). Many of them were proteins involved in folding and maturation like pdi-1, ER resident chaperones, bip-1, grp 78, and several heat shock proteins. These genes were induced in response to increased up regulation of CWDE influenced by 2-DG. These data suggested that the secretory pathway in this fungus regulates increased lignocellulase synthesis and secretion by up regulating the ER stress responsive proteins to reduce ER load (Figures 6, 7). Protein disulphide isomerase (PDI) is one of the major ER foldases. The enzyme catalyses the formation and rearrangement of disulphide bridges in proteins during folding. The pdi gene was upregulated in treated condition.and its expression level was qPCR (Figure 8B). The differential expression levels of several chaperons and binding protein were also upregulated (Figure 6), although the expression of bZIP transcription factor and HAC 1 showed minor increase (Figures 6, 8B).

Secondary metabolites are involved in intracellular, intercellular and interspecific interactions*. A*. is known to produces a wide variety of secondary metabolites, and this motivated us to find the molecular basis of this production by *de novo* transcriptomics. We identified 30 core genes related to secondary metabolism including 12 polyketide synthase (PKSs), four putative PKS-like enzymes, seven fatty-acid synthases, (Monooxygenase FAD-binding protein etc. Supplementary Table 1; Octave et al., 2006).

### Other artifacts of 2-DG treatments in the culture media

#### 2-DG reduced cell viability followed by cell death

A large number cell repair, senescence, p38, mapk, glutathione-s-transferase, pho81 was induced by 2-DG (Supplementary Table 4). This is in concurrence to the condition induced by carbon starvation in fungi (Emri et al., 2004; Pócsi et al., 2004; Katz et al., 2006; Szilágyi et al., 2013). This data suggest that macrophagy is a part of late response to 2-DG in *A. malaysianum* as observed in old cultures by gradual pigmentation and mycelia fragmentation. However, a more detailed study is required to further establish this effect. The production of cell wall hydrolyzing enzymes may also trigger cell death process in old carbon starved cultures (Emri et al., 2013). In our study, there was up regulation of cell wall integrity pathway genes such as WSC, SNF-1, and other map kinases that are usually present in carbon starvation or depletion like condition (Table 3). The degradation of glutathione is significant, because glutathione is utilized as an energy source, supports intracellular amino acid transport and protects cells against ROS. Interestingly, 2DG resulted in up regulation of the expression of glutathione-s-transferase but down regulation of superoxide (Figure 6).

#### Expanded transporter gene families in treated condition

In plant pathogens, cellular transporters are responsible not only for export of compounds involved in pathogenesis and virulence, but they also may play an essential role in protection against plant defense compounds (e.g., secondary metabolites) during pathogenesis, possibly by exporting host-derived antimicrobial compounds out of the cell (Pao et al., 1998; Schoonbeek et al., 2002). The transportation system is involved in uptake of essential nutrients and ions, excretion of metabolic end products, and deleterious substances, and communication between cells and the environment. Transporters comprised of 5.3% of our classified transcripts (the second largest cluster after carbohydrate biosynthesis). Specific transporters elevated such as those of ABC, hexoses, solute, sugar:H+ symporter, MFS transporters. A total of 46 genes encoding transporters were identified in the merged transcriptome highest order of MFS transporters. MFS transporters are involved in the transport of monosaccharides, oligosaccharides, inositols, drugs, amino acids, nucleosides, organophosphate esters, Krebs cycle metabolites, and a large variety of organic and inorganic anions and cations.Compared to the control, a significant increase in MFS transporters was observed in the transcriptome of treated. The gene number of sugar transporter (SP) family of MFS subfamily was higher indicating the uptake of more carbohydrate-produced nutrients. Comparative analysis with other fungi revealed that the MFS alpha-glucoside transporter, MFS multidrug transporter is overrepresented, suggesting export of more metabolism products in the extracellular. Presence of a high affinity nicotinate permease that catalyzes nicotinic acid (vitamin B3) uptake, vitamin H transporter, putative vitamin b6 transporter, bsu1 protein, vitamin H transporter 1/biotin symporter vht1, reflecting that might be dependent from the host plant for vitamin supply (Table 3).

### Validating RNAseq results with qPCR techniques

Seven differentially expressed transcripts, of which most of them were related to metabolism (Hexokinase, Fructose bisphosphate, Isocitrate lyase, Transaldolase), protein folding (Protein disulfide isomerase, HAC) and cell wall degrading enzyme (β-glucosidase) were selected for validating the results obtained RNAseq analyses using qPCR (Table 5). In this analysis, a comparison has been made for the expression of the chosen genes between the Control (absence of 2-DG) and treated (presence of 2-DG) as illustrated in Figure 8. This study showed that most of the gene related to metabolism and protein folding were found to be upregulated in 2-DG treatment compared to the control, which showed no such up-regulation.

**Table 5:** **List of genes selected for differential expression analysis and the oligonucleotides used in this study**.

**Gene ID**	**Gene**	**Primers**	**Mean fold change in expression level**	**Regulation**
AC	Actin (Reference gene)	Fw: GCTGCCCTCTTATCGACAA		
		Rv: TGTGATGCCAGATCTTCTCCAT		
FRU	Fructose-1,6-bisphosphatase	Fw: CCCAGCTTGTCATCACCATG	Control: 1	UP
		Rv: TCTTCCCAGTACATCGCGTT	Treated: 3.4032	
ISO	Isocitrate Lyase	Fw: GTTCATGGCCCAGATGTTCC	Control: 1	DOWN
		Rv: CTTGGTCAGCTTCATGACGG	Treated: 0.3956	
PDI	Protein disulfide isomerase	Fw: ACCTCGAGAGCTTGACCAAA	Control: 1	UP
		Rv: GGGAGCAGTGAAAGCAACAA	Treated: 2.391	
BGL	β-glucosidase	Fw: AGGTCTGCCTCGTCTTCCTGAAG	Control: 1	UP
		Rv: AGGATGGCAGTCACATTGGGGTG	Treated: 4.521	
ALD	Aldolase	Fw: CCTTCACCTCATCGAGCTCT	Control: 1	UP
		Rv: GGTGGAGAACATGAGGGTCA	Treated: 3.182	
HAC	Hac	Fw: TAATACGACTCACTATAGGGC	Control: 1	SAME
		Rv: GCGCTCTAGAACTAGTGGATCC	Treated: 1	
HXK	Hexokinase	Fw: CAAAGTGACAGTGGGTGTGG	Control: 1	UP
		Rv: GCCAGGTCCTTCACTGTCTC	Treated: 1.4032	

*Sample spreadsheet of data analysis using the 2^−ΔΔCT^ method. The fold change in expression of the target gene relative to the internal control gene (–actin). The samples were analyzed using qPCR and the Ct data were imported into Microsoft Excel. The mean fold change in expression of the target calculated where ΔΔC_T_ (C_T_,_Target_—C,_Actin_) treated—(C_T, Target_—C,_Actin_) control*.

We examined transcript levels of β-glucosidase differentially expressed gene through qPCR, which were also found to be differentially expressed at 2-D proteomic analysis (Figure 2B). The expression levels of two well-known UPR maker genes such as PDI and HACA was determined using qPCR and was found that though there was increased expression of Protein disulfide isomerase the expression of HAC remained unaltered. The results obtained from qPCR data are concordant with our transcriptomics data, confirming the reliability of the results with minimum discrepancies.

## Discussion

### Cellulose degrading enzyme production in response to 2-deoxyglucose are results of shunting of catabolite repression

*A. malaysianum* has an innate ability to secrete lignocellulose-degrading enzymes in the extracellular medium (Table 1A). The higher level of production of these enzymes makes *A. malaysianum* an industrially important strain for biofuel production that can be rapidly exploited. *In vitro* addition of 2-DG in cellobiose medium resulted in further increase of β-glucosidase and endoglucanase activity by several folds (Table 1B). It may be proposed that such rise in β-glucosidase results from the combined action of two mechanisms acting simultaneously; (i) Shunting down of the catabolite repression of the cell by glucose anti-metabolite 2-DG in the medium; (ii) cellobiose induces lignocellulolytic enzymes. The non- metabolizable glucose analog 2-DG has been used extensively to induce catabolite repression in bacteria and fungi (Kornberg and Lambourne, 1994). Moreover, the use of 2-deoxyglucose (2-DG), as an antimetabolite has been routinely practiced for selection of the carbohydrate degrading enzyme hyper-producing mutants. The addition of 2-DG to cells causes a glucose starvation-like response, inhibiting growth and reducing viability even in the presence of abundant glucose. 2-DG is taken up and converted to 2-deoxyglucose-6-phosphate (2-DG-6P). However, the absence of a hydroxyl group on C-2 prevents the further catabolism of 2DG-6P by phosphogluco-isomerase. Accumulation of 2DG-6P in the cytoplasm result in inhibition of growth by repressing early glycolytic enzymes, thereby inhibiting glycolysis. Whether these are the only means by which 2-DG short-circuits normal glucose utilization needs to be explored further.

Exogeneous addition of 2-DG in a growth media containing a sole carbon source extended the lag phase, and inhibited growth in fungi (Zhu et al., 2015). In presence of preferred carbon source (glucose), sugar uptake and utilization was unaffected and maximum growth was observed in 48 h in absence as well as in presence of 2-DG. However, in cellobiose medium 2-DG caused delayed growth, indicating that 2-deoxyglucose led to CCR of cellobiose utilization (Figures 1A,B). Similar result was observed in *Thermoanaerobacterium aotearoense* where 0.5 mg/ml of 2-DG strongly inhibited growth (Zhu et al., 2015). The CCR is generally controlled by the second messenger cyclic adenosine 3',5'-monophosphate (cAMP), which has a fundamental role in global gene regulation (Lin et al., 2013). Catabolite repression by 2-DG was not reversed by adding exogenous cAMP (10 mM; Figure 1C). Similar studies on another Ascomycetous *Aspergillus nidulans* also corroborates our observation of no CCR relief in presence of cAMP (Borgia and Sypherd, 1977; Lee et al., 1996).

Since 2-DG hampers microorganism's growth, it is widely used as an inducer of CCR (Zhu et al., 2015). We added different concentration of 2-DG (0.25–1 mg/ml) to two different carbon source (1% cellobiose and glucose media) to detect the CCR by monitoring the culture growth. 2-DG delayed growth in cellobiose but not in glucose cultures. In glucose medium maximum growth (measured as mycelia dry weight) was observed in 48 h both in absence and presence of 2DG (Figure 1A). The growth rate measured in absence of 2-DG was 0.31 mg/g and 0.22 mg/g at 1 mg/ml concentration. In cellobiose medium maximum growth in absence of 2DG was observed at 60 h (0.271 mg/g) and was lagged upto 36 h with increasing concentration of 2DG (0.25–1 mg/ml). The growth density was also visibly reduced with increasing 2DG concentration (Figures 3A,B). Addition of 10 mM cAMP showed no effect on the growth delay caused by 2-DG in cellobiose medium (Figure 1C), unlike in *E. coli* where exogenous addition of cAMP in the medium provided CCR relief (Ullmann and Monod, 1968; Isaacs et al., 1994). This proves that in *A. malaysianum*, 2-DG-induced CCR is cAMP-independent.

### Release of extracellular β-glucoside in response to 2-DG is involved in cellular signaling

In filamentous fungus, signal transduction pathways have been found to be largely associated with cell wall weakening (Wang et al., 2014). In *N. crassa* addition of 1 mM cellobiose and 2-deoxy-glucose inhibited the secretion of β-glucoside. Although the actual mechanism behind this was not discussed it was hypothesized that 2-DG was nearly as effective as glucose in blocking induction. Segregated and pellet like hyphae is probably the consequence of hyphae fragmentation due to production of cell wall hydrolytic enzymes like endochitinase and endo β-1,3 glucanases (Szilágyi et al., 2012, 2013) (Figures 3A–D).

Since 2-DG is a non-metabolizable glucose analog and also a glycosylation inhibitor, it markedly reduced the amount of cell wall components resulting in declining dry cell mass and progressive fragmentation of the hyphae in *A. malaysianum* (Figures 3E–H, 10). Cell wall of *S. cerevisae* grown in presence of 0.2%, 2-DG contained less glucose and mannose compared to the normal cell (McCartney et al., 2014). In the present study, we gained a more complex view of the changes taking place in the cell wall homeostasis. We detected the induction of several genes encoding hydrolytic enzymes such as chitinase, glucan β-1,3-glucosidase (Table 1B) and down regulation of chitin synthase in 2-DG treated condition (Figure 6; Table 4). Similar induction of several genes encoding cell wall hydrolyzing enzymes (chitinase, α and β-glucanases and mannanases) has been detected in carbon starved cultures of *Aspergillus niger* (Nitsche et al., 2012). Up regulation of putative β-glucosidase encoding genes might also contribute to the degradation of the fungal cell wall, as observed by enhanced extracellular β-glucosidase activity in presence of 2-DG (Figure 2; Table 3; Szilágyi et al., 2010).

Interestingly the degree in depression in growth rate and cell wall composition was dose dependent. In the culture with 0.025% 2-DG, the decrease in cell viability was much lesser compared to 0.05 and 0.1% 2-DG concentration (data not shown). In both 0.05 and 0.1% 2-DG cell lysis took places from late exponential phase to early stationary phase. 2-DG (0.1% w/v) added at inoculation time to cultures of *Aspergillus* prevents α-l, 3-glucan, α-l, 3-glucanase, and cleistothecium formation. 2-DG given after α-1,3-glucan synthesis, inhibits a-l, 3-glucanase and glucan breakdown partially in *Aspergillus* (Lee et al., 1996). By RNA sequencing and secretome analysis, we identified seven fungal hydrophobin, five chitinase, two endochitinase, one chitinase 18-18, proteases, one chitinasechi18-5, one Chitinase precursor, one Chitin-binding type1 and 2 chitin synthase genes in the transcriptome of *A. malaysianum* (Supplementary Tables 1, 2).

### Distinctive expansion of gene families associated with plant cell wall degradation, secondary metabolism, and nutrient uptake in the transcriptome of *A. malaysianum*

The expanded CAZyme arsenals of *A. malaysianum* were similar to those of *Eutypa lata, Thielavia terrestris*, and *Colletotrichum sp*. (Table 6). Interestingly, these fungi are known to be pathogen on host plants and have been isolated as endophytes from other sources also. Glycoside hydrolases (GHs) emerges as a large family under differentially expressed gene category. Unlike pathogens that thrive on pectin-rich tissue (Zhao et al., 2013), which possesses high numbers of pectolytic enzymes, *A. malaysianum* showed a wider array of enzymes that target cellulose and hemicellulose. This includes endo-β-1,4-cellulases (GH5), β-glucosidases (GH3), xyloglucan transglucosylase/hydrolases (GH16), and β-xylosidases (GH43). We found significant expansion of genes coding for CAZymes containing the CBM1 domain, a carbohydrate-binding module. CBM1 strongly binds to crystalline cellulose and that is required for full activity of fungal cellulases similar to the findings from *E. lata* that has a similar CBM1gene family expansion pattern (Klosterman et al., 2011).

**Table 6:** **CAZymes showing maximum similarity with Eutypa lata UCREL1, Thielavia terrestris NRRL, and ***Colletotrichum sp*****.

**ID**	**ORGANISM**	**DESCRIPTION**
comp146372_c0_seq1	*Eutypa lata* UCREL1	beta-glucosidase 2
comp133529_c0_seq1	*Eutypa lata* UCREL1	GH 55
comp3205_c1_seq1	*Eutypa lata* UCREL1	alpha-n-arabino-furanosidase
comp8979_c0_seq1	*Eutypa lata* UCREL1	GH 17 protein
comp563262_c0_seq1	*Eutypa lata* UCREL1	endo-beta-glucosidase
comp524152_c0_seq1	*Eutypa lata* UCREL1	galactan-beta-galactosidase
comp524152_c0_seq1	*Eutypa lata* UCREL1	alpha-glucuronidase
comp150388_c0_seq1	*Thielavia terrestris* NRRL	GH 7
comp114670_c0_seq1	*Thielavia terrestris* NRRL	GH 3
comp89_c0_seq1	*Thielavia terrestris* NRRL	polysaccharide lyase family 4 protein
comp606705_c0_seq1	*Thielavia terrestris* NRRL	GH 1
comp117007_c0_seq1	*Colletotrichum sp*.	GH 92
comp116499_c0_seq1	*Colletotrichum sp*.	GH 76
comp110262_c0_seq1	*Colletotrichum sp*.	GH 31
comp523355_c0_seq1	*Colletotrichum sp*.	endoglucanase
comp136830_c0_seq1	*Colletotrichum sp*.	GH 92
comp136956_c0_seq1	*Colletotrichum sp*.	alpha-glucosidase
comp3208_c0_seq1	*Colletotrichum sp*.	Acetyl xylan esterase precursor
comp542888_c0_seq1	*Colletotrichum sp*.	pectinesterase
comp180674_c0_seq1	*Colletotrichum sp*.	Exo-polygalacturonase

P450s have a broad spectrum of functions in fungi, from housekeeping activities, such as synthesis of essential membrane lipids, to synthesis of secondary metabolites, and detoxification of xenobiotic compounds (Chen et al., 2014). Differences in the number of genes belonging to the different P450 classes were observed among species, particularly between the Ascomycetes and Basidiomycete (Brakhage, 2013). *A. malaysianum* harbors high copies of antifungal, antioxidant, monooxygenases and cytochomeP450 superfamily genes including the genes responsible for the production of rare alkaloid—Loleine (Wang et al., 2008; Table 3). Three different types of CYP families were identified in *A. malaysianum*: cytochrome c, b560, and the P450. The high diversity of secondary metabolites is related to the diversity of the CYP genes. For example, the 219 CYP genes in the *Ganoderma lucidum* genome resulted in a large number of different secondary metabolites (Yu et al., 2012).

**Figure 9:**
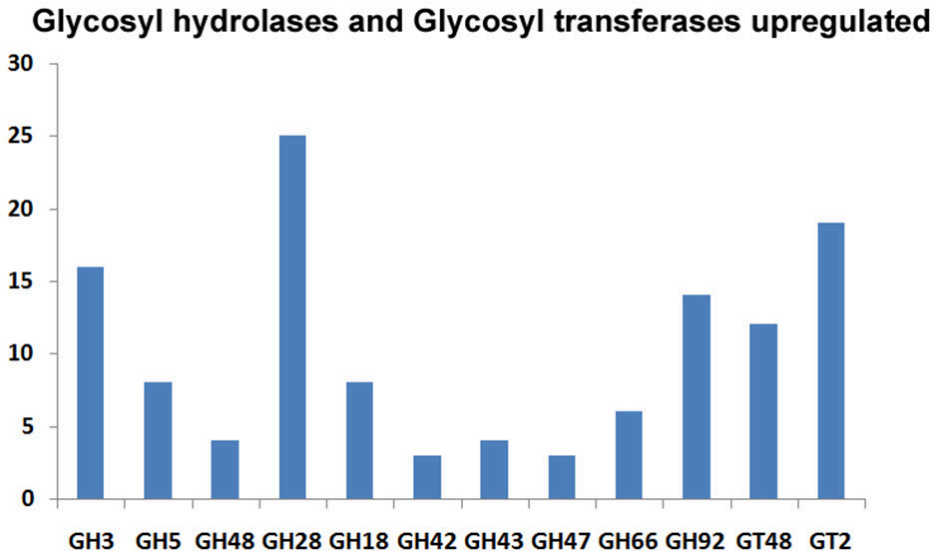
**Classification of the upregulated CAZymes under 2-DG treatment**.

The CAZymes-encoding genes that are significantly up-regulated in treated as compared to the control condition include many glycosyl hydrolases (GH classes; Cantarel et al., 2009) and glycosyl transferases: GH5 (8), GH3 (16), GH28 (25), GH18 (8) and GH66 (6), GT48, GT2 (19), GT17 (5), GT22 (5), and GT55 (5) (Figure 9). Polygalacturonases belonging to Glycosyl hydrolases family 28 was highly upregulated. Eight genes encoding different GH28 family pectinolytic enzymes (pectinesterase, polygalacturonase, pectate lyase proteins) were expressed.

### Metabolic pathways involved in carbon flow cycle are perturbed in response to 2-DG

To gain an insight into the altered metabolic status imposed by 2-DG, four different enzymes involved in the interlinked pathways of glycolysis (hexokinase), gluconeogenesis (fructose-bisphosphatase), Krebs' cycle (isocitrate lyase, malate dehydrogenase), and glyoxalate cycle (isocitrate lyase) were ascertained and compared in qPCR (Figure 8B; Table 5).

**Figure 10:**
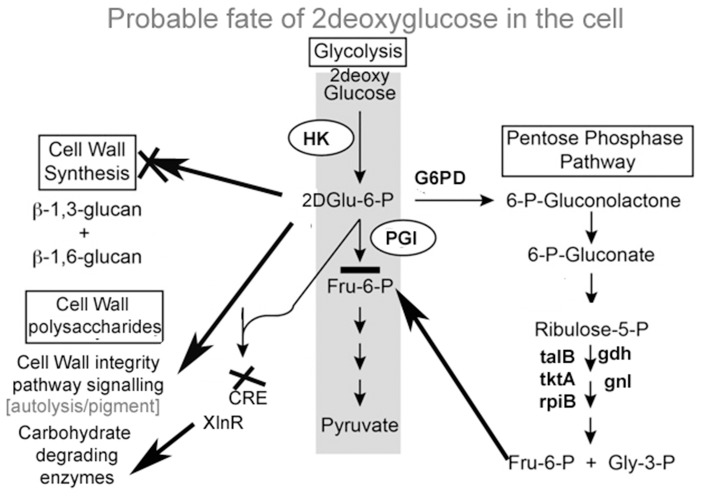
**Schematic representation of different pathway activated under 2-DG**.

Pathways associated with Hexokinase, Transaldolase, F-6-phosphate, and glucose-6 phosphate dehydrogenase and alcohol dehydrogenase production were induced whereas Isocitrate lyase and Phosphogluco-isomerase were suppressed (Figures 8A,B). The activity and expression fold of these marker metabolic enzymes was quantified by enzymatic assay and also determined by qPCR data reinforcing our suggested carbon flow cycle (Livak and Schmittgen, 2001; Figure 10). The accumulation of non metabolizable 2DG-6-P in the cytoplasm forced the cell to recover from the glucose sink by entering into Pentose phosphate Pathway and gluconeogenesis pathway. This leads to up regulation of several metabolic enzymes like transaldolase (talB), transketolase (tktA), fructose bis phosphate (fbp), glucose 1 dehydrogenase (gdh), gluconolactonase (gnl), ribose 5 phosphate isomerase (rpiB; Figure 6).

CreA/Cre1- knock out mutants display severe phenotypic changes such as reduced growth, abnormal hyphal morphology and sporulation. Up regulation of different genes linked to carbohydrate metabolism (e.g., glycoside hydrolases and glycoside transferases), cellular transport, signaling molecules like MAPK and SNF2 as well as certain chaperons and heat shock protein that is induced by several stresses, including glucose limitation (Portnoy et al., 2011). It may be presumed that 2-DG works in a manner homologous to these Cre mutants creating a similar environment and displays similar traits (Figure 10).

## Conclusion

Tricking *A. malaysianum* into producing industrially important enzymes with 2-Deoxy D-Glucose treatment is a novel way to increase commercial usability of this fungi. The ability of *A. malaysianum* to produce hemicellulolytic enzymes renders it an attractive organism for the production of fuels and renewable energy. We investigated 2-DG induced carbon metabolism and carbohydrate degrading enzymes secretion in this organism in order to better understand the regulatory mechanism of this non metabolized sugar and how to manipulate it. Transcriptomics study and our experimental data indicate that *A. malaysianum* is regulated in a CCR independent mechanism in presence of 2DG. The induced synthesis of secondary metabolites, fatty acid and lipids, and proteins associated with cell wall degrading enzymes demonstrated the benefits of addition of 2-DG in the culture medium. 2-DG induces a carbon starving like environment causing increased production of proteins and extracellular enzymes. As a result, endoplasmic reticulum activity increases considerably leading in further up regulation of transporters, disulfide isomerases, and binding proteins. Plethora of secretory enzymes linked to carbohydrate degradation and several metabolites having economic values can further be explored for commercialization.

## Data availability

Sequence data is publicly available in Genbank with SRA id SRR746797. The Bioproject id is PRJNA186407 and Biosample id is SAMN01923006.

## Author contribution

SK and ST conceived the project. SM and SK designed the project. SM conducted the experiment. AP, MC, and ST analyzed the data. SM, ST, and MC. wrote the manuscript.

## Funding

ST would like to acknowledge DBT - Ramalingaswamy fellowship for supporting this work. SM would like to acknowledge CSIR, Govt of India for funding (Award no. 31/2 (901)/2012-EMR-I). MMC. would like to acknowledge DST for fellowship.

### Conflict of interest statement

The author declares that the research was conducted in the absence of any commercial or financial relationships that could be construed as a potential conflict of interest.

## Supplementary material

Supplementary Table 1 **Parsed output of blast results of merged transcripts (control + treated) against Non Redundant database**.

Supplementary Table 2 **Catalog of secretory proteins under 2-DG treated and control conditions**.

Supplementary Table 3 **Differential expression analysis of 2-DG treated samples with control**. Positive values indicate up-regulation under 2-DG and negative values indicate down-regulation. CAZY annotation, interproscan, and blast annotations are provided alongside.

Supplementary Table 4 **Pathway analysis and KO IDs of the entire merged (Control + treated) transcripts**.

## Abbreviations

2-DG: 2-deoxy D-glucose
KEGG: Kyoto Encyclopedia of Genes and Genomes
GO: Gene ontology
CAZY: Carbohydrate Active enzymes
UPR: Unfolded protein response
qPCR: Quantitative Real time PCR
GPI-anchored: Glycophosphatidylinositol anchored protein
cAMP: Cyclic adenosine monophosphate
ORF: Open reading frame.
